# Mapping connectivity fingerprints for presurgical evaluation of temporal lobe epilepsy

**DOI:** 10.1186/s12883-021-02469-1

**Published:** 2021-11-10

**Authors:** Jacint Sala-Padro, Júlia Miró, Antoni Rodriguez-Fornells, Xavier Rifa-Ros, Gerard Plans, Mila Santurino, Mercè Falip, Estela Càmara

**Affiliations:** 1 Epilepsy Unit, Hospital de Bellvitge, Barcelona, Spain; 2 Cognition and Brain Plasticity Unit, Bellvitge Biomedical Research Institute (IDIBELL), L’Hospitalet de Llobregat, 08097 Barcelona, Spain; 3 Department of Cognition, Development and Educational Science, Campus Bellvitge, University of Barcelona, L’Hospitalet de Llobregat, 08097 Barcelona, Spain; 4 Catalan Institution for Research and Advanced Studies, ICREA, Barcelona, Spain

**Keywords:** Epilepsy, Surgery, Temporal lobe, Biomarker, Prognosis

## Abstract

**Background:**

Surgery may render temporal lobe epilepsy (TLE) patients seizure-free. However, TLE is a heterogenous entity and surgical prognosis varies between patients. Network-based biomarkers have been shown to be altered in TLE patients and hold promise for classifying TLE subtypes and improving pre-surgical prognosis. The aim of the present study is to investigate a network-based biomarker, the weighted degree of connectivity (wDC), on an individual level, and its relation to TLE subtypes and surgical prognosis.

**Methods:**

Thirty unilateral TLE patients undergoing the same surgical procedure (anterior temporal resection) and 18 healthy controls were included. All patients were followed-up in the same center for a mean time of 6.85 years and classified as seizure-free (SF) and non seizure-free (non-SF). Using pre-surgical resting state functional MRI, whole brain wDC values for patients and controls were calculated. Then, we divided both temporal lobes in three Regions-of-interest (ROIs) -mesial, pole and lateral- as these areas are known to behave differently in seizure onset and propagation, delimiting different TLE profiles. The wDC values for the defined ROIs of each individual patient were compared with the healthy group.

**Results:**

After surgery, 14 TLE patients remained SF. As a group, patients had higher wDC than controls in both the temporal pole (*p* < 0.05) as well as in the mesial regions (*p* < 0.002) of the to-be-resected temporal lobe. When comparing between SF and non-SF patients, a step-wise binary logistic regression model including all the ROIs, showed that having an increased wDC of the temporal pole (*p* < 0.05) and the mesial area (*p* < 0.05) of the to-be-resected temporal lobe was associated with seizure freedom long-term after surgery.

**Conclusions:**

This study provides a network-based presurgical biomarker that could pave the way towards personalized prediction. In patients with TLE undergoing anterior temporal resections, having an increased wDC at rest could be a signature of the epileptogenic area, and could help identifying those patients who would benefit most from surgery.

## Background

Surgery for drug-resistant temporal lobe epilepsy (TLE) has proven to be an effective treatment to cure or reduce the frequency of disabling seizures. Surgery for TLE resects or disconnects the epileptogenic zone, the region where the seizure is generated [1]. However, around 50% of patients who undergo surgery for epilepsy fail to remain long-term seizure free [2].

In order to evaluate the candidacy for surgery in TLE patients, a complete pre-surgical evaluation of patients is performed using multiple investigations [video-electroencephalography (VEEG), neuropsychological studies, structural Magnetic Resonance Imaging (MRI), Positron Emission Tomography (PET) and Single Proton Emission Computerized Tomography (SPECT)]. This allows assessment for whether the abnormalities observed are regionally confined to the unilateral temporal region, and therefore, whether the patient is a good candidate for surgery. Predicting which patients will benefit most from surgery has proven to be difficult, as TLE is a heterogenous disorder, representing different entities [3, 4], and with different patterns of relapse after surgery [5].

In recent years, approaching TLE as a brain network disease has been a framework hypothesis that improved the understanding of electrophysiological and imaging findings in patients with epilepsy [6, 7]. Indeed, there are neuroimaging methods which have shown promising results in determining the outcome of surgery by analyzing brain networks, but are not yet in widespread use [8, 9]. One of these methods is resting-state functional MRI (rs-fMRI), which provides good spatial and temporal information of the epileptic brain, thereby allowing brain network analysis. In patients with focal epilepsy, a shift from a small world topology network seen on healthy controls to a more ordered network has been described: key findings include higher clustering and lesser integration in the interictal epileptic brain [10]; also, peaks of local connectivity have been found around the epileptogenic area [11]. Furthermore, previous studies have demonstrated how different subtypes of TLE display different network connectivity, measurable by rs-fMRI [12, 13]. Relating to surgical prognosis, an increase in hubness of the thalamus, by measuring its degree of connectivity, could be associated with relapse after surgery [14]. An interesting measure in this growing field that allows the study of local functional synchronization is the degree of centrality (DC) [15]. This measure considers both the local functional connectivity and the global network alterations, and evaluates the number of strongly correlated links to a given area [16].

Previous factors relating to poor seizure outcome on TLE are the presence of an abnormality in the pathology specimen after surgery, and its detection with pre-surgical MRI [17]; the presence of secondary generalized seizures [18] and the involvement of structures outside the mesial temporal lobe in seizure activity, such as the temporal neocortex and the temporal pole [19], or extratemporal structures [4]. The above mentioned factors related to seizure relapse after surgery do also relate to the extent of TLE network disease [3, 20]. Moreover, different pathological substrates express different network abnormalities [13]. In this sense, rs-fMRI may be capable of detecting focal brain connectivity and network alterations in the temporal lobe of patients with TLE, which would in turn help in the localization of the epileptogenic area, the definition of the subtype of TLE by assessing the connectivity of different temporal lobe areas and, ultimately, the relationship to prognosis after surgery.

Our aim is to analyze a series of patients with TLE undergoing standard anterior temporal resection (ATLR), including the information from rs-fMRI analysis for the characterization of the epileptic network, with the goal of predicting the extension of the disease and detecting which patients will benefit most from surgery, through an individual based approach. To this end, we study the degree of connectivity as a proxy of the focal connectivity within the temporal lobe, accounting for both its local and whole brain connectivity, and its relationship with the surgical outcome.

## Methods

### Patients

We recruited 33 consecutive patients with TLE who underwent a pre-surgical evaluation between 2009 and 2014 and were considered candidates for surgery. Patients underwent a complete work-up with VEEG, standard neuropsychological evaluation, structural MRI, and, in some cases, FDG-PET and ictal/interictal SPECT. They were diagnosed by epileptologists according to clinical, EEG and imaging criteria. All patients underwent a 3 T MRI with a resting state fMRI sequence. We also scanned 18 healthy volunteers with the same protocol, matched for age, gender, handedness and years of education.

We included 30 patients into the study; two patients finally declined surgery and one patient was discarded due to erroneous data acquisition. Of these 30 patients, 17 were women (56.7%) and 15 had left temporal epilepsy. All patients underwent the same procedure (ATLR) performed by the same surgeon. All pathology samples were processed in our hospital and examined by the same pathologist. After surgery, patients were followed-up on a three to six monthly basis, specifically interrogating for seizure relapse, for a mean time of 82.3 months (6.85 years), with a range of 39 to 118 months.

The normative group was comprised of 18 healthy participants who had no record of neurological illnesses or psychiatric disorders, and were matched for age, gender, handedness and years of education. See Table 1 for demographic details of the patients and healthy group samples. This study was approved by the Ethical Committee of Hospital of Bellvitge.

**Table 1:** Sociodemographic and clinical characteristics of TLE patients

		TLE Patients	Seizure Free	Non-Seizure Free	***p***-value	Healthy participants
*N*		30	14	16		18
Age		49.8 ± 11.1	50.7 ± 10.9	48.9 ± 11.6	*n.s.*	49.50 ± 11.92
Sex(f)		17(56.7%)	9(52.9%)	8(47.1%)	*n.s.*	11 (61%)
Left TLE		15(50%)	7(46.7%)	8(53.3%)	*n.s.*	
Age onset seizure		15.2 ± 11.6	11.7 ± 10.1	18.3 ± 12.3	*n.s.*	
Duration of epilepsy		27.4 ± 15.4	32.7 ± 13.9	22.7 ± 15.5	*n.s.*	
Febrile seizures		6(20%)	2(33.3%)	4(66.7%)	*n.s.*	
Secondary generalisation		22(75.9%)	10(45.5%)	12(54.5%)	*n.s.*	
ILAE Outcome [21]	1		14 (46.6%)			
	2			3(10%)		
	3			9(30%)		
	4			2(6.6%)		
	5			2(6.6%)		
	6			–		
Lesion MRI		25 (83.3%)	13(53%)	12(48%)	*n.s.*	
Pathology	HS	21(70%)	11(52.4%)	10(47.6%)	*n.s*	
	Tumor	1(3.3%)	1(100%)	–		
	Heterotropia	1(3.3%)	1(100%)	–		
	Normal	5(16.7%)	–	5 (100%)		
	Dual (HS + cavernoma/ tumor)	2(6.7%)	1(50%)	1(50%)		

Data presented as *mean* ± *standard error* and *N (%patient)*. *N* is detailed in individual cells where differing. Age, Age of onset seizure and duration of epilepsy are given in years. The *p*-values refer to independent two-tailed t tests between seizure free and non-seizure free patients

*N* number of participants; *f* females; *ILAE* International League Against Epilepsy; *HS* Hippocampal Sclerosis; *n.s* non significant (*p* > 0.05)

### MRI data acquisition

Patients and healthy participants underwent a pre-operative whole-brain structural MRI scans using a 3.0 Tesla Siemens Trio MRI. A 32-channel phased-array head coil system was used to acquire high-resolution T1-weighted images (slice thickness = 1 mm; no gap; number of slices = 240; TR = 2300 ms, TE = 3 ms, matrix = 256 × 256; FOV = 244 mm; voxel size 1x1x1 mm). Resting state fMRI data were collected using a single-shot T2*-weighted gradient-echo EPI sequence (slice thickness = 4 mm; no gap; number ofslices = 32, interleaved order; TR = 2000 ms; TE = 29 ms; flipangle = 80°; matrix = 80 × 80; voxel size = 3 × 3 × 4 mm^3^, 110 volumes).

### Resting-state functional connectivity analysis

Resting-state functional connectivity preprocessing was carried out using the pipelines implemented in Data Processing Assistant for Resting-State fMRI [22] (DPARSF, http://rfmri.org/DPARSF), which is based on Statistical Parametric Mapping (SPM, http://www.fil.ion.ucl.ac.uk/spm) and the toolbox for Data Processing & Analysis of Brain Imaging [23] (DPABI, http://rfmri.org/DPABI) running on MATLAB (v17.a, Mathworks, Natick, MA). The preprocessing steps included the exclusion of the first five volumes, slice timing adjustment and realignment for head motion correction. Coregistration between the functional and the structural T1-weighted image, segmentation of the T1-weighted image into different tissues, DARTEL normalization of the functional and structural images to the MNI space using the parameters derived from the segmentation of the T1-weighted image. Subsequently, the covariates including the white matter signal, cerebrospinal fluid signal, Friston 24 motion parameters and polinominal trend, were regressed out from the time series of every voxel. Furthermore, the BOLD signal was filtered in order to reduce potential effects of low-frequency drift and high-frequency physiological noise with a typical temporal bandpass (0.01–0.1 Hz). Finally, the automated anatomical labeling (AAL) template of Tzourio-Mazoyer et al*.* [24] was used to parcellate registered fMRI time series into specific masks and to create the study regions of interest (ROIs) for the to-be resected (ipsilateral) temporal lobes. Specifically, the hippocampus, amygdala and parahippocampus masks were used to define the mesial ROI; the superior and inferior masks of the temporal pole for the temporal pole ROI, and the superior, middle and inferior temporal gyrus masks to define the lateral temporal lobe ROI. These ROIs were selected as these areas are crucial in seizure relapse as well as defining the TLE subtype [4, 19].

Weighted Degree of Centrality (wDC) maps, restricted to the defined ROIs, were computed by using the REST toolbox, using a similar approach to that shown by Buckner et al. and Zuo et al. [25, 26], as previously described [27–29]. Specifically, first, for each voxel, Pearson’s correlation coefficient between the time series of that voxel and all other voxels were calculated, resulting in a connectivity map that represents all other voxels that are correlated with the selected voxel. Then, this correlation map was thresholded at *r* > 0.25. The weighted sum of previous significant positive correlations was calculated to yield the weighted wDC at the selected voxel. This process was repeated for each voxel in the brain to produce a voxel-wise whole-brain map of the weighted degree of centrality. Then, the individual weighted degree of centrality maps were standardized across all voxels by converting to z-scores [25, 26, 30]. Finally, before statistical analysis, the individual maps of degree centrality were spatially smoothed using a 8 mm Gaussian kernel.

### Statistical analyses

Statistical analysis of group demographics, outcome-based classification, and clinical data was performed in SPSS (v.22, SPSS Inc., Chicago, USA). Chi square and two-tailed independent samples Student’s t-test were used to describe clinical and sociodemographic differences between groups. Logistic regression was used to classify the patients based on the surgical outcome.

Second level models of voxel-based wDC neuroimaging data were performed using SPM12 software (http://www.fil.ion.ucl.ac.uk/spm/). Specifically, in order to explore the normative wDC pattern, the individual voxel-wise wDC maps of the healthy group were entered into a second-level analysis using a one-sample t-test. Then, in order to identify group differences in voxel-based DC maps, both healthy participants vs. TLE patients and seizure-free vs. non seizure-free patients were compared using a two-sample t tests by entering the corresponding voxel-wise wDC maps. Whole-brain level significant results were identified at *p* < .005 and corrected for multiple comparisons at cluster-level (*p* < .05), with a minimum cluster size of 20 contiguous voxels. When examining seizure-free vs. non seizure-free patients, an exploratory threshold was applied (*p* < .005 at voxel-level, uncorrected). This approach is designed to minimize both Type I and Type II errors in a way that facilitates replication of results across future studies [31]. Anatomical and cytoarchitectonic areas were identified using the Automated Anatomical Labeling Atlas [24] included in the xjView toolbox (http://www.alivelearn.net/xjview8/).

In order to identify individual regionally specific effects in DC, individual voxel-wise wDC maps were compared between each subject and the normative group within the defined ROIs using a two-tailed Crawford’s modified *t*-tests [32, 33], as done in previous studies [34, 35]. This test is specifically designed to compare a patient to a small sample of control participants [36]. Differences were considered statistically significant when *p* < .005. As a next step, for each ROI a binary map of significant wDC was calculated, setting all connections below the significant threshold to zero while setting all remaining connections to 1. Then, one binary variable was created to indicate whether any of the voxel of the selected ROI showed significant differences in the wDC. In addition, a confounding variable was created accounting for the number of significant voxels included in the selected ROI. This process was repeated for each ROI using MATLAB in-house code (MATLAB R2017a, MathWorks, Natick, MA).

In order to evaluate whether the selected variables could be used to classify the patients based on the surgical outcome, a logistic regression was performed. More specifically, a binary step-wise backward logistic regression [37] between both the levels of wDC burden and the number of significant voxels in the different ROIs and, presence of recurrence during follow-up (i.e. seizure-free versus non seizure-free) was assessed. The Hosmer-Lemeshow C statistic was used to calculate the goodness of fit of the logistic model [37]. Beta parameters (B), Nagelkerke’s and Cox-Snell’s R^2^ values for each model were specified. In addition, the receiver operating characteristic (ROC) curve was plotted, and the area under the curve (AUC) was calculated as a summary statistic for the prognostic model. One way of interpreting the AUC is as the probability that given any 2 participants randomly selected, one who becomes Seizure Free and one who relapse after surgery, the model would assign a higher probability of Seizure Free than the non-Seizure Free.

## Results

Of the 30 patients, 14 remained completely seizure free (SF) and 16 were not seizure free (non-SF) at the end of the follow-up. Comparing SF and non-SF, there were no significant differences in gender (64.3% vs 50%, *p* = 0.431), history of febrile seizures (14.3% vs 25%, *p* = 0.657), history of generalized tonic-clonic seizures (71.8% vs 80%, *p* = 0.590), presence of an MRI lesion (92.9% vs 81.3%, *p* = 0.602) and presence of hippocampal sclerosis (HS) on tissue sample (85.7% vs 65.8%, *p* = 0.399). SF and non-SF patients had the same epilepsy duration (35.16 vs 27.16, *p* = 0.180). For the clinical characteristics of the sample, see Table 1.

**Fig. 1:**
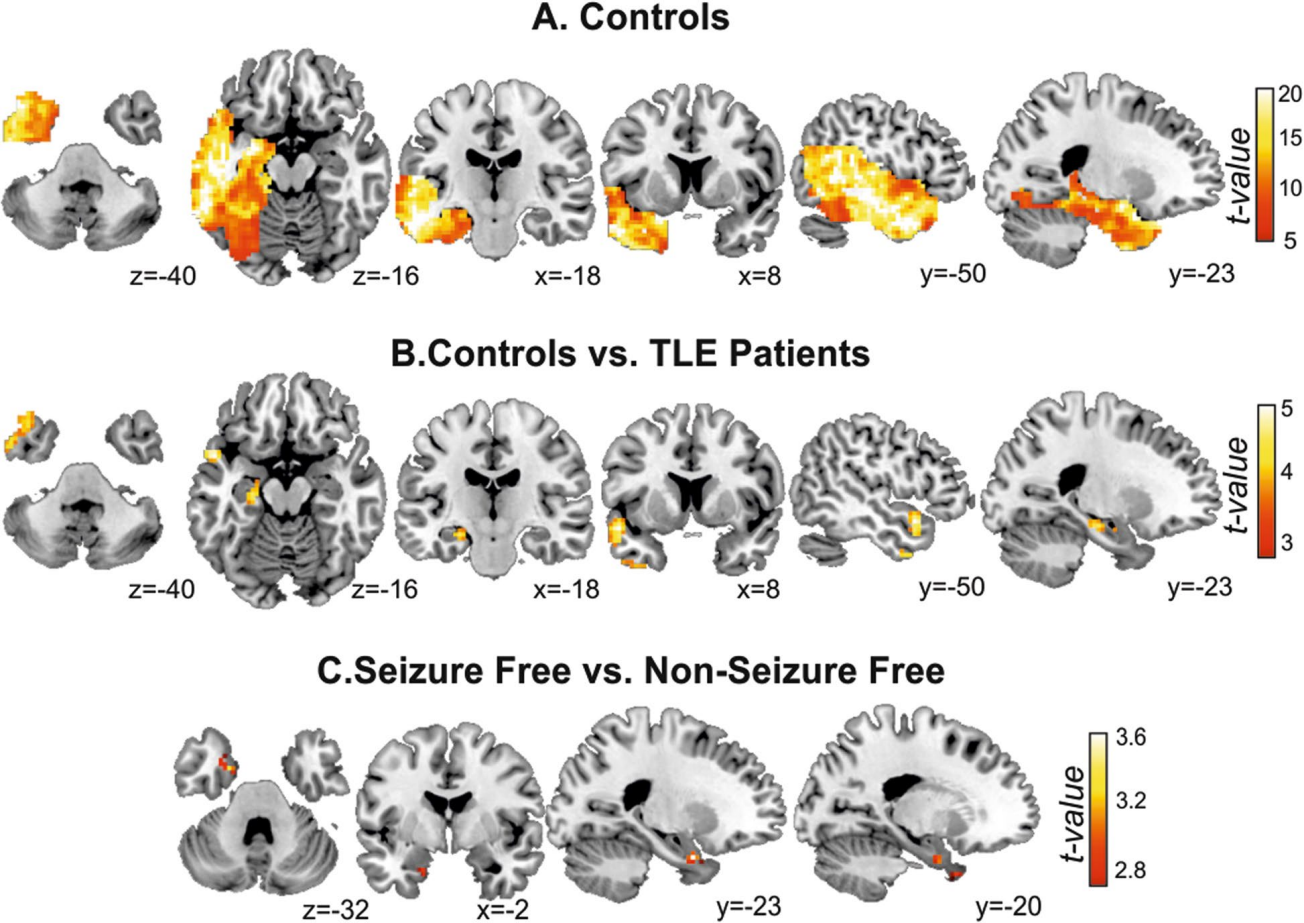
Functional connectivity results at whole-brain level in contols (**A**) and between group differences (**B**-**C**). Slice position is labeled in Montreal Neurological Institute coordinates

We first carried out a voxel-based analysis restricted to the affected TLE regions in the different groups. Indeed, an exploratory analysis to the normative group showed significantly high levels of wDC in an extended pattern, highlighting cortical regions in the lateral temporal lobe, especially in the inferior and middle temporal gyri (See Fig. 1A). Moreover, the comparison between Healthy participants and TLE patients revealed higher wDC in both the superior (t = 5.02, x = − 51, y = 9, z = − 15, *p* < 0.009, FDR-corrected at cluster level) and the inferior (t = 4.07, x = − 54, y = 0, z = − 36, *p* < 0.012, FDR-corrected at cluster level) temporal pole structures, as well as in hippocampal regions (t = 4.23, x = − 24, y = − 18, z = − 15, *p* < 0.002, FDR-corrected at cluster level) (See Fig. 1B). In addition, after surgery, SF patients presented a reduced level in wDC levels in comparison to Non-SF patients in parahippocampal regions extended to the temporal pole (x = − 21, y = 3, z = − 27, *p* < 0,001 uncorrected) (See Fig. 1C). However, this difference was not statistically significant when correcting for multiple comparisons.

Then, in order to further explore individual differences associated with specific patterns in DC, single subject comparison to the normative group and a logistic regression analysis were investigated. We obtained the individual wDC for the different ROIs described above by comparing each patient with the set of healthy participants. By binarizing the presence or absence of increased wDC according to a threshold, we obtained a number of patients with increased wDC for each ROI (Table 2). A majority of patients had an increase of wDC of the lateral temporal lobe (96.7% for the ipsilateral side, 93.3% for the contralateral). Approximately half of the patients had increased wDC of the temporal pole (56.7% ipsilateral, 46.7% contralateral), and a minority of patients had increased wDC of the mesial area (23.3% ipsilateral, 16.8% contralateral) when compared to controls. Due to the fact that the majority of patients in our sample presented HS (70% of the total), we also looked at the increase in wDC for each ROI in this subgroup of patients (Table 2).

**Table 2:** Pattern of weighted Degree of Centrality for each Region of interest

	All patients (*n* = 30)		SF (*n* = 14)		Non-SF (*n* = 16)	
	Increased DC	Non-increased DC	Increased DC	Non-increased DC	Increased DC	Non-increased DC
Mesial – ipsilateral	7 (23.3%)	23 (76.7%)	6 (42.9%)	8 (57.1%)	1 (6.3%)	15 (93.8%)
Temporal pole – ipsilateral	17 (56.7%)	13 (43.3%)	12 (85.7%)	2 (14.3%)	5 (31.3%)	11 (68.8%)
Lateral temporal - ipsilateral	29 (96.7%)	1 (3.3%)	14 (100%)	*Nil*	15 (93.8%)	1 (6.3%)
Mesial – contralateral	5 (16.7%)	25 (83.3%)	4 (28.6%)	10 (71.4%)	1 (6.3%)	15 (93.8%)
Temporal pole – contralateral	14 (46.7%)	16 (53.3%)	8 (57.1%)	6 (42.9%)	6 (37.5%)	10 (62.5%)
Lateral temporal - contralateral	28 (93.3%)	2 (6.7%)	14 (100%)	*Nil*	14 (87.5%)	2 (12.5%)
	HS patients (*n* = 23)		SF (*n* = 12)		Non-SF (*n* = 11)	
	**Increased DC**	**Non-increased DC**	**Increased DC**	**Non-increased DC**	**Increased DC**	**Non-increased DC**
Mesial – ipsilateral	7 (30.4%)	16 (69.6%)	6 (50%)	6 (50%)	1 (9.1%)	10 (90.9%)
Temporal pole – ipsilateral	13 (56.5%)	10 (43.5%)	10 (83.3%)	2 (16.7%)	3 (27.3%)	8 (72.7%)
Lateral temporal - ipsilateral	22 (95.7%)	1 (4.3%)	12 (100%)	*Nil*	15 (90.9%)	1 (9.1%)
Mesial – contralateral	4 (17.4%)	19 (82.6%)	4 (33.3%)	8 (66.7%)	*Nil*	11 (100%)
Temporal pole – contralateral	10 (43.5%)	13 (56.5%)	8 (66.7%)	4 (33.3%)	2 (18.2%)	9 (81.8%)
Lateral temporal - contralateral	22 (95.7%)	1 (4.3%)	12 (100%)	*Nil*	10 (90.9%)	1 (9.1%)

Number of patients with increased wDC for each ROI, for the whole sample and for patients with HS (including patients with dual pathology). Obtained by comparison against controls, a threshold of *p* < 0.005 was applied for considering the wDC of a ROI to be increased. SF – Seizure free. HS – Hippocampal sclerosis

**Fig. 2:**
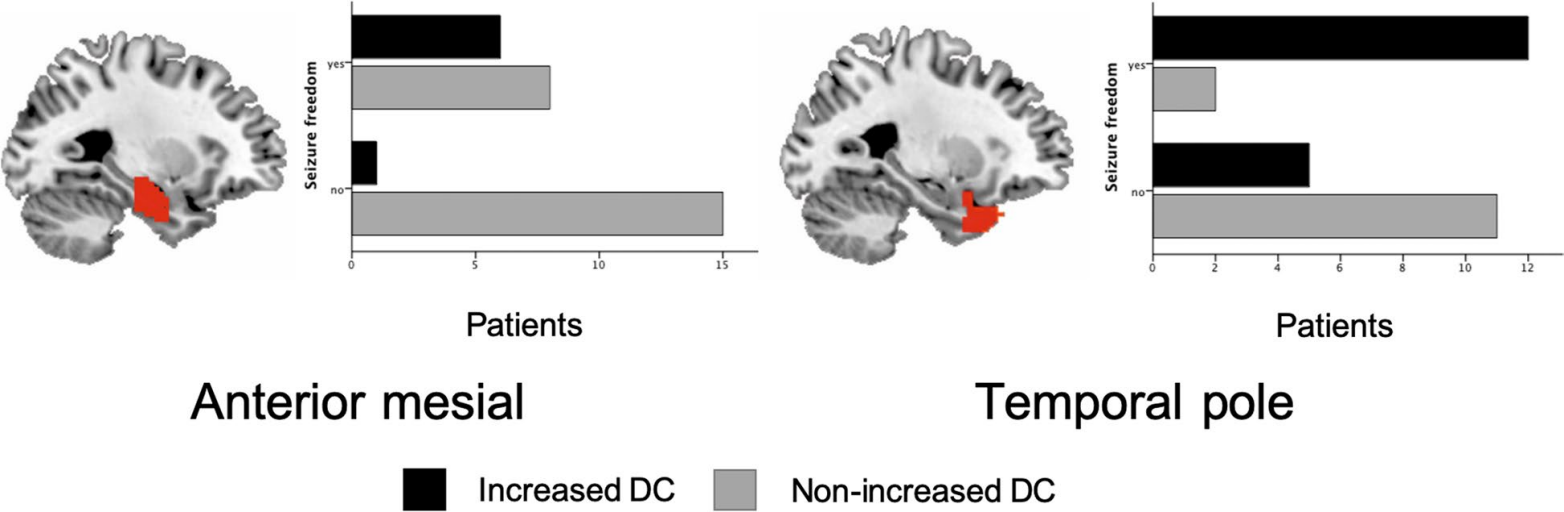
Illustration of the wDC distribution in seizure free and non-seizure free patients in anterior mesial and temporal pole ROIs. Both areas were significantly associated with seizure freedom in the logistic regression model

**Fig. 3:**
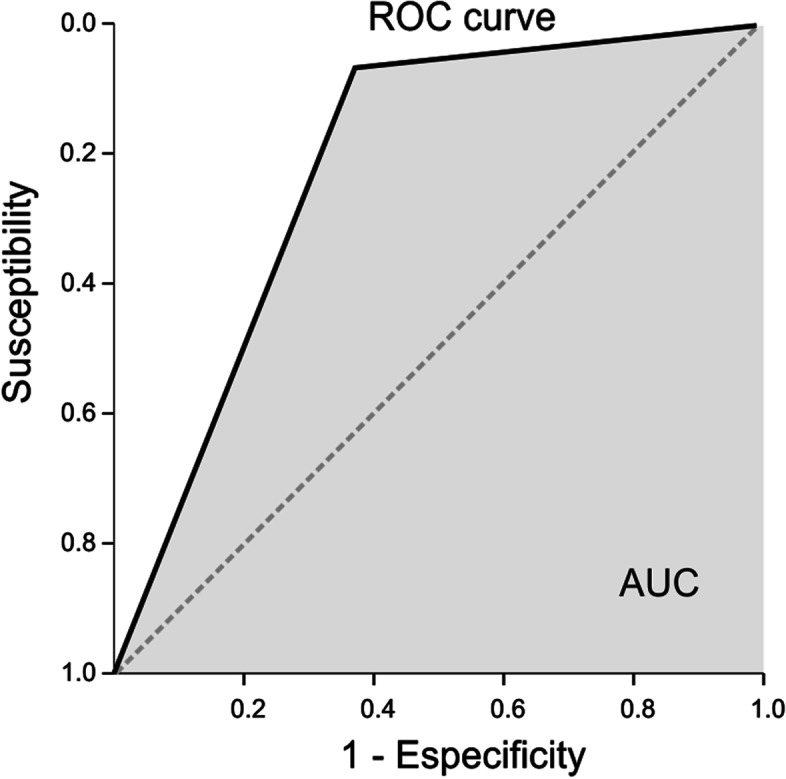
Receiver Operating Characteristic (ROC) curve used to classify the patients based on the surgical outcome and the connectivity parameters extracted from the selected ROIs (Regions of Interest). AUC = Area under the curve

In a step-wise binary logistic regression model including all the ROIs, having an increased wDC of the temporal pole (*p* < 0.05) and of the mesial area (*p* < 0.05) was associated with seizure freedom long-term after surgery for the whole TLE patient sample (Fig. 2, Table 3). Notice that the number of affected voxels within each ROI was not associated with the surgery outcome in any of the regions. The model correctly classified 76.7% of the patients, 92.9% in the SF group, 62.5% in the non-SF group. Finally, the ROC curve was calculated; an AUC of 0.777 was founded (Fig. 3).

**Table 3:** Model Goodness-of-fit and Regressors Significance of Logistic Regression

Model goodness-of-fit	R^2^ Cox-Snell	R^2^ Negalkerke	X^2^	P-value	Hosmer-Lemeshow
	0.39	0.53	15.19	0.001	0.3
**Regressors**	B	SE		P-value	
Mesial wDC	−2.88	1.47		0.05	
Temporal pole wDC	−2.91	1.17		0.013	

*R2* coefficient of determination; *X2* chi-square; *B* coefficient for the predictor; *SE* standard error of the coefficient for the predictor; *wDC* weighted Degree Centrality

## Discussion

Our findings reveal crucial network-related biomarkers extracted from individual and quantitative rs-fMRI measures related with surgical outcome in a set of TLE patients with a long-term follow-up. Specifically, at individual level, increases in the local connectivity of key structures within the epileptogenic network in TLE, such as the temporal pole and the mesial area, were related to surgical outcome in our series of patients with TLE undergoing anterior temporal resection. The patient cohort had a mean follow-up of more than 6 years, which means that most of the SF patients will remain cured [2]. This information could be readily available as part of the pre-surgical counselling and evaluation.

DC is a connectivity measure of intrinsic focal connectivity that accounts for highly connected areas, both locally and globally, highlighting those regions that act as main stations for information processing (i.e., hubs) that form connections with other segregated networks [26]. In our study, we parcellated the temporal lobe into mesial, temporo-lateral and temporo-polar areas. These three regions are known to be implicated in the genesis of temporal lobe seizures and, moreover, relate with both TLE subtypes and surgical prognosis, as highlighted in previous studies [5, 19]. In order to focus on individual based data, we used a voxel-to-voxel approach and compared each patient with a set of healthy participants. We found that increased DC of the temporo-polar and the antero-mesial regions were related with the odds of remaining seizure free long-term after the surgery.

Although there is a great overlap between the different connectivity patterns associated with TLE, there is some degree of differentiation. Patients with TLE display increased local connectivity, consistently reported in the epileptogenic area, with decreased long-range connectivity [38]. The measurement of peaks of local connectivity has been related to the epileptogenic area, and its resection has translated into improvement of whole brain network topology [11]. Outside the temporal lobe, an increase in connectivity of the thalamus related to poorer seizure prognosis in patients with TLE, probably relating to a widespread network disease [14]. Conversely, reductions in long range connectivity measures with increases in local connectivity, when confined to the resected to be temporal lobe, were related to surgical prognosis [39]. Using a measure of focal voxel-wise connectivity, we investigated the degree of connectivity in the to-be-resected temporal lobe, parcellated in three areas that both relate to the subtype of TLE and are known to have implications in surgical prognosis. Specifically, an increased wDC related to a better prognosis, which may be attributed to a peak of strong local connectivity, i.e., a marker of the epileptogenicity of the region, and therefore indicate the appropriateness of its resection. TLE with or without HS comprises different entities, with variable extra-mesial involvement, and different patterns of seizure relapse after surgery [3]. Notwithstanding, measuring local increased connectivity on an individual level could be a helpful biomarker in evaluating the connectivity within the to-be-resected temporal lobe, which evidence suggest to be relevant in surgical prognosis. Noteworthy, our wDC measure does not distinguish between short- or long-range connectivity, which could provide additional information on the network connectivity of the temporal lobe in TLE, but should otherwise be evaluated with other network-related parameters.

In our sample, patients with an increase in temporo-polar wDC compared with controls had higher odds of becoming seizure-free. The temporo-polar cortex is known to be involved in seizures arising from the temporal lobe [19]. In a study using independent component analysis from rs-fMRI data that compared patients with mesial TLE due to different etiologies with healthy controls, found that those patients with mesial TLE patients demonstrated increased connectivity between the temporal pole and the post-central gyrus [40]. Our sample consists mainly of patients with clinical and EEG findings suggestive of mesial TLE, and the majority of patients had HS on the tissue sample (Table 1). However, not all patients with HS had increased temporo-polar wDC, and the same proportion of patients without HS also had increased temporo-polar wDC (Table 2). In a study using intracranial recordings, a key role of the temporal pole cortex was reported in TLE seizures, in close relation with the hippocampus [19]. Patients with quicker involvement of the temporal pole had better surgical outcome than those with late involvement. We therefore hypothesized that, this early and consistent involvement of the temporal pole could imply increase network abnormalities in this area. As mentioned before, peaks of local connectivity are found around the structures implicated in seizure generation, the epileptogenic area [11]; therefore, an increased wDC of the temporal pole could be a network biomarker that relates to its epileptogenicity; that is, its implication in seizure generation, a reported finding known to especially relate to a better surgical prognosis.

The other major finding is that an increased mesial wDC was also related to a better prognosis, with only one patient relapsing after surgery. This mesial wDC increase was seen only in our patients with HS on pathology (Table 2). Previous reports using rs-fMRI have shown that patients with HS displayed increased connectivity of the mesial structures, and this is distinctive from non-lesional MRI TLE patients [12]. In our sample, all the patients with HS and increased connectivity remained seizure free except one. Interestingly, the relapsed patient that demonstrated an increased wDC on the mesial structures was an individual with dual pathology. As HS may be a heterogenous group of diseases [41], identifying markers of good surgical response is of paramount interest for the clinician; we related our finding of increased wDC to HS with better prognosis. This finding needs to be reported with longer series in order to study whether an increased wDC of the anterior mesial area in patients with HS represents a subgroup of patients with better prognosis.

Assessing the extension of the epileptogenic zone and identifying the subtype of TLE is crucial to decide the candidacy for a temporal lobe resection, the need of intracranial evaluation and establishing a prognosis. One of the most important factors for a surgical procedure in TLE is having a lesion on MRI. However, there is still a proportion of patients with lesions on MRI that relapse, as well as patients with normal MRI that benefit from surgery. Beyond the MRI being lesional or not lesional, studies using PET and SPECT have rendered different patterns that relate to different prognosis, helping decision making [42, 43]. From our study, the role of rs-fMRI could help identify key network abnormalities that may improve the assessment of prognosis before the surgery.

**Fig. 4:**
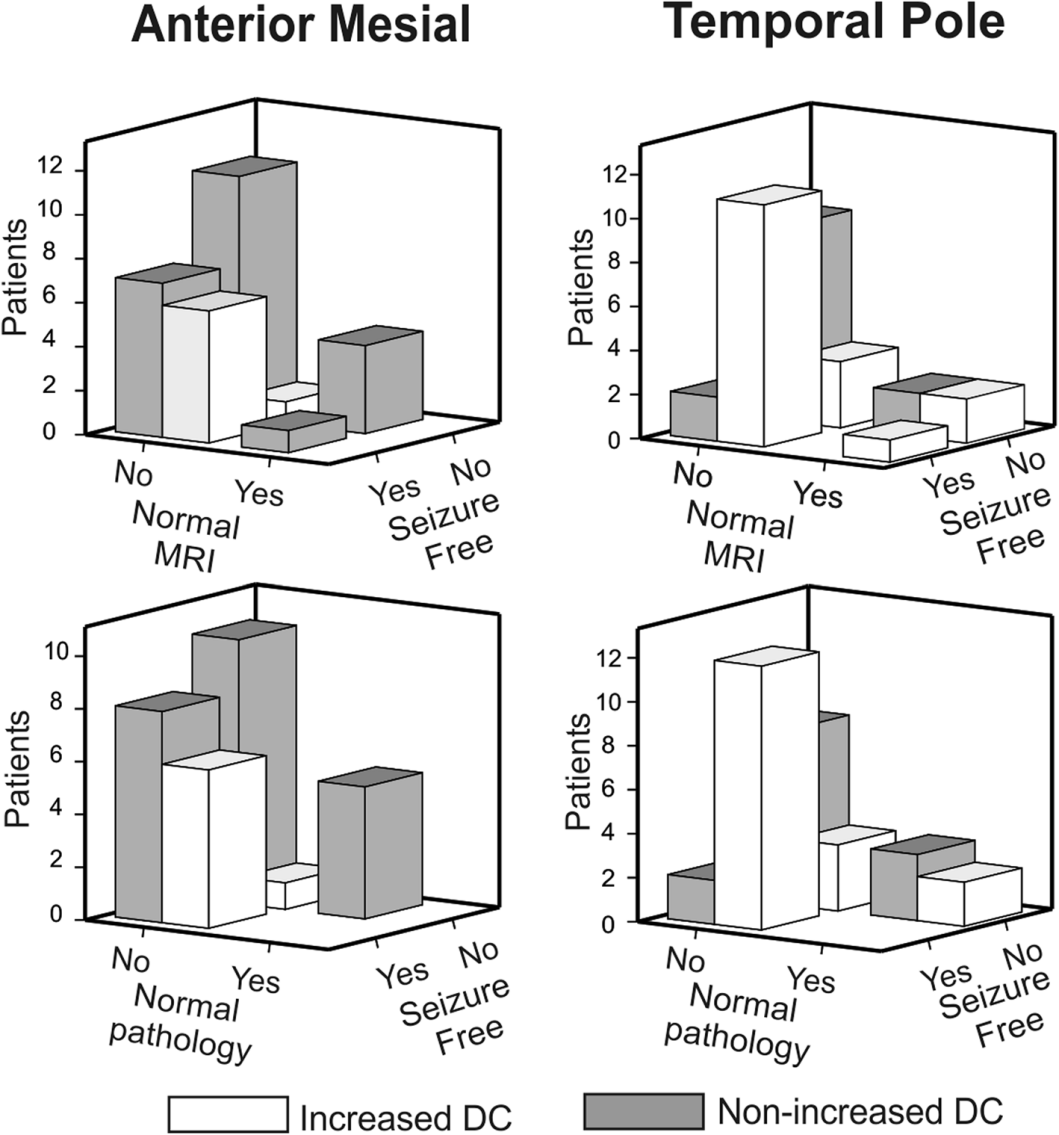
Distribution of patients with normal MRI and normal pathology, relation with seizure freedom and increased wDC. Left panel represents the distribution in the anterior mesial region; right panel shows the temporal pole of the epileptogenic brain size

Several considerations form our study must be taken. First, as we studied our own cohort of TLE surgical patients, our sample consists mostly of MRI lesional patients, as patients with HS formed a large proportion of the total sample. While the use of rs-fMRI would not change decision making, using our findings as reported could provide extra information on prognosis at the individual level. On the other hand, we report few TLE patients with normal MRI and normal pathology, which are presumably the ones in need of more pre-surgical tools to help decision-making. These patients could benefit most of new individual biomarkers complementary to the ones currently in use during pre-surgical evaluation. Even though in our small sample of normal MRI and normal pathology the findings reported were significant (Fig. 4), longer series are needed to validate the usefulness of rs-fMRI. As mentioned, TLE is a heterogeneous disease, with many factors influencing the surgical outcome. We strove to control for clinical factors in SF and non-SF groups (no differences on history of febrile convulsions or generalized tonic-clonic seizures), and we report a similar number of patients with lesional MRI and HS on pathology on both SF and non-SF groups. Finally, due to the fact that all patients underwent anterior temporal lobectomies performed by the same surgeon, we did not control the tissue resected. Further studies should account for the resected tissue after the surgical procedure, by measuring the wDC of the actual resected area and investigating network changes after the resection.

## Conclusion

Elucidating the neurobiology of the different profiles within TLE is of paramount relevance for the stratification of surgical outcomes. Fingerprinting brain connectivity biomarkers of the to-be-resected temporal areas could contribute to the establishment of a prognosis prior to surgery and provide more promising personalized healthcare.

## Data Availability

The data that support the findings of this study are available on reasonable request from the corresponding author, EC.

## Abbreviations

TLE: Temporal lobe epilepsy
VEEG: Videoelectroencephalography
MRI: Magnetic Resonance Imaging
PET: Positron Emission Tomography
SPECT: Single Proton Emission Computerized Tomography
Rs-fMRI: Resting State Functional Magnetic Resonance Imaging
(w)DC: (weighted) Degree Centrality
ATLR: Anterior Temporal Lobe Resection
SF: Seizure free
Non-SF: Non seizure free
AAL: Automated Anatomical Labeling
ROI: Region Of Interest
TLE-HS: Temporal lobe epilepsy with Hippocampal Sclerosis
